# A Descriptive Analysis of Pediatric Transports Throughout the U.S. Indo-Pacific Command

**DOI:** 10.1093/milmed/usaa506

**Published:** 2021-07-01

**Authors:** Ashley E Sam, Mitchell T Hamele, Renée I Matos, Angela M Fagiana, Matthew A Borgman, Joseph K Maddry, Steven G Schauer

**Affiliations:** San Antonio Uniformed Services Education Consortium, Department of Pediatrics, Brooke Army Medical Center, JBSA-Fort Sam Houston, TX 78234, USA; 59th Medical Wing, JBSA-Lackland, TX 78236, USA; Department of Pediatrics, Tripler Army Medical Center, Honolulu, HI 96859, USA; Uniformed Services University of the Health Sciences, Bethesda, MD 20814, USA; San Antonio Uniformed Services Education Consortium, Department of Pediatrics, Brooke Army Medical Center, JBSA-Fort Sam Houston, TX 78234, USA; 59th Medical Wing, JBSA-Lackland, TX 78236, USA; Uniformed Services University of the Health Sciences, Bethesda, MD 20814, USA; San Antonio Uniformed Services Education Consortium, Department of Pediatrics, Brooke Army Medical Center, JBSA-Fort Sam Houston, TX 78234, USA; Uniformed Services University of the Health Sciences, Bethesda, MD 20814, USA; San Antonio Uniformed Services Education Consortium, Department of Pediatrics, Brooke Army Medical Center, JBSA-Fort Sam Houston, TX 78234, USA; Uniformed Services University of the Health Sciences, Bethesda, MD 20814, USA; 59th Medical Wing, JBSA-Lackland, TX 78236, USA; Uniformed Services University of the Health Sciences, Bethesda, MD 20814, USA; US Army Institute of Surgical Research, JBSA-Fort Sam Houston, TX 78234, USA; 59th Medical Wing, JBSA-Lackland, TX 78236, USA; Uniformed Services University of the Health Sciences, Bethesda, MD 20814, USA; US Army Institute of Surgical Research, JBSA-Fort Sam Houston, TX 78234, USA

## Abstract

**Background:**

The U.S. Indo-Pacific Command (INDOPACOM) has over 375,000 military personnel, civilian employees, and their dependents. Routine pediatric care is available in theater, but pediatric subspecialty, surgical, and intensive care often require patient movement. Transfer is frequently performed by military air evacuation teams and intermittently augmented by civilian services. Pediatric care requires special training and equipment, yet most transports are staffed by non-pediatric specialists. We seek to describe the epidemiology of pediatric transport missions in INDOPACOM.

**Methods:**

A retrospective review of all patients less than 18 years old transported within INDOPACOM and logged into the Transportation Command Regulating and Command and Control Evacuation System (TRAC2ES) database from June 2008 through June 2018 was conducted. Data are reported using descriptive statistics. Patients were categorized into four age groups: neonatal (<31 days), infant (31-364 days), young children (1 to <8 years), and older children (8-17 years).

**Results:**

During the study period, 687 out of 4,217 (16.3%) transports were children. Median age was 4 years (interquartile range 6 months to 8 years) and 654 patients (95.2%) were transported via military fixed-wing aircraft. There were 219 (31.9%) neonates, 162 (23.6%) infants, 133 (19.4%) young children, and 173 (25.2%) older children. Most common diagnoses encountered were respiratory, cardiac, or abdominal, although older children had a higher percentage of psychiatric diagnoses (28%). Mechanical ventilation was used in 118 (17.2%) patients, and 75 (63.6%) of these patients were neonates.

**Conclusions:**

Within TRAC2ES, nearly one in six encounters were patients aged <18 years, with neonates or infants representing nearly one of three pediatric encounters. Slightly more than one in six pediatric patients required intubation for transport. The data suggest the need for appropriately trained transport teams and equipment be provided to support these missions.

## INTRODUCTION

**FIGURE 1.**
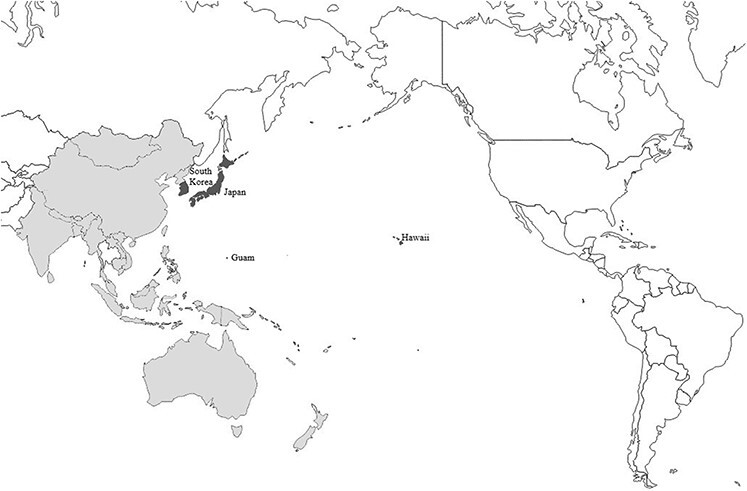
Map demonstrating area controlled by the U.S. Pacific Command. Countries/states highlighted in dark grey are major origin/destination sites for pediatrics transports.

The U.S. Pacific Command (PACOM), now known as the U.S. Indo-Pacific Command (U.S. INDOPACOM) as of June 2018, is the largest of the six geographical combatant commands, encompassing 36 nations and employing approximately 375,000 U.S. military and civilian personnel (Fig. 1).^1–3^ At several duty locations throughout INDOPACOM, both military and civilian DoD employees are accompanied by dependents. While routine pediatric care is available at these duty stations, pediatric subspecialty care and intensive care often require treatment at a larger medical treatment facility (MTF) or civilian institutions not available within the area of operations.

The Armed Forces work jointly using the Transportation Command (TRANSCOM) and U.S. Air Force’s Air Mobility Command to manage the medical transport of patients across the globe.^4^ All patient transports on aircraft will have an air evacuation (AE) team that is comprised of flight nurses and medics.^5^ Patient acuity may necessitate AE team augmentation with Critical Care Air Transport Teams (CCATTs). These teams are comprised of a physician (typically an emergency medicine physician, anesthesiologist, or critical care physician), a nurse (most often emergency room or intensive care unit [ICU] trained), and a respiratory therapist.^6^ For neonatal transport, a neonatal intensive care unit (NICU) nurse and medic from the NICU, as well as a neonatologist if patient acuity requires direct physician oversight, may augment the AE team.

The transport of pediatric patients within INDOPACOM poses unique challenges since no formal pediatric CCATT exists. Neonatal and pediatric transport teams within the United States transport the majority of their patients by ground and most often travel less than 250 miles between destinations as the transfers are usually from a community hospital to a larger receiving center in a nearby major city.^7^ Additionally, many facilities across the United States have either unit-based or dedicated pediatric transport teams regardless of patient acuity.^7,8^ In contrast, due to the location of military bases across INDOPACOM, transport to definitive pediatric care often requires the use of aircraft and involves transoceanic flights that may be several thousand miles in length with transport times of more than 6 hours. Furthermore, while there is neonatal transport team capability, there is no dedicated or specifically trained pediatric transport team in INDOPACOM.

Although the military maintains the TRANSCOM Regulating and Command and Control Evacuation System (TRAC2ES) to coordinate military medical transports, there are limited data surrounding military transport of pediatric patients worldwide and no published data detailing the epidemiology of pediatric transports throughout INDOPACOM. Understanding the epidemiology surrounding pediatric transport missions is crucial for logistical preparations and developing effective training to prepare providers on AE teams and CCATTs to provide optimal care to pediatric patients.

### Goal of This Study

The study sought to describe pediatric movements throughout the PACOM area of operations using patient movement encounters within the TRAC2ES.

## METHODS

The 59th Medical Wing regulatory office reviewed protocol FWH20180147E and determined it was exempt from Institutional Review Board oversight. Only de-identified data were obtained.

Data were collected retrospectively by way of data request from TRAC2ES, an electronic platform that coordinates medical transport of all DoD patients worldwide.^9^ Data entered in TRAC2ES include patient demographics, primary diagnosis, origin, destination, evacuation priority level, and a free text space for patient history or clinical course.

All non-active duty patients under 18 years logged in TRAC2ES who were transported either to or from the INDOPACOM region between June 2008 and June 2018 were included. The initial search returned 698 patients. Nonhuman movements (e.g., military working dogs) were excluded resulting in the inclusion of 687 patient transports in the final dataset.

Data were analyzed based on gender, transport status, military versus civilian transport, ground versus air transport, origin and destination site, disease category, and need for mechanical ventilation. Transport status, disease category, and ventilation status were further evaluated by age using groupings of <31 days (neonates), 31 days to <1 year (infants), 1 to <8 years (young children), and 8 to <18 years (older children and adolescents). A three-tier system was used to categorize transport status in accordance with the definitions used by USAF AE teams and the flight surgeon within INDOPACOM.^5^ Transports were categorized as “urgent” requiring transport within 24 hours to save life, limb, or major organ, “priority” leaving on the next scheduled mission or sooner without tolerance for delays, typically within 1-7 days, and “routine” leaving on the next scheduled mission, but may be rescheduled as needed. Twelve descriptive disease categories for the data were agreed upon by the authors and chosen based on the major body system and/or primary concern involved in the patient’s primary diagnosis listed in TRAC2ES. The category of “other” encompassed medical, obstetrical, and surgical diagnoses that did not otherwise fit into our established categories. The following terms were searched in the free text space to evaluate for ventilator status: intubate, intubated, ventilator, endotr-acheal tube.

Statistical analysis was performed using Microsoft Excel (version 10, Redmond, Washington) and JMP Statistical Discovery from SAS (version 13, Cary, NC). Data are reported using descriptive statistics, reporting categorical variables as numbers with percentages and ordinal variables as medians with interquartile ranges.

## RESULTS

There were 4,217 patient encounters in INDOPACOM from June 2008 through June 2018 entered into TRAC2ES. Of these, 687 (16.3%) were pediatric patients. The majority of patients transported were less than 1 year old with 219 (31.9%) patients being <31 days old and 162 (23.6%) patients being 31 days to 1 year old. Most patients (95.2%, *n* = 654) were transported by military transport teams with an overwhelming majority being transported by aircraft (99.7%, *n* = 685.) The majority of transports originated in Japan (55.3%, *n* = 380) and concluded in Hawaii (51.1%, *n* = 351) (Table I).

**TABLE I.** Patient Demographics

Demographics	*n* (%)	
Age		Median (interquartile range)
<31 days	219 (31.9)	7 days (2-15.3 days)
31 days to <1 year	162 (23.6)	2.5 months (1.7-3.3 months)
1 to<8 years	133 (19.4)	3 years (2-5 years)
> 8 years	173 (25.2)	13 years (11-15 years)
Total	687	4 years (0.5-8 years)
Patient’s gender		
Male	392 (57.1)	
Female	295 (42.9)	
Transportation method		
Military	654 (95.2)	
Civilian	33 (4.8)	
Air transport	685 (99.7)	
Ground transport	2 (0.3)	
Origin country		
Japan	380 (55.3)	
Guam	118 (17.2)	
South Korea	19 (2.8)	
United States-Hawaii	127 (18.5)	
Other	43 (6.3)	
Destination country		
Japan	88 (12.8)	
Guam	26 (3.8)	
United States-Hawaii	351 (51.1)	
United States- continental United States	153 (22.3)	
Other	69 (10.1)	

Of all patients, 12.8% (*n* = 88) were transported in “urgent” status where transport is typically within 24 hours in order to save life, limb, or major organs (Table II).^5^ Fifty-eight of the 88 (62.5%) urgent evacuations recorded were patients 30 days of age or less. As the patient cohort age increased, the percentage of urgent transports decreased with patients over 8 years of age only making up 7.9% of all urgent transports (*n* = 7, 4.1% of all patients over 8 years).

**TABLE II.** Evacuation Status, Diagnosis, and Ventilator Status by Age

Age	<30 days	31 days-1 year	1-<8 years	>8 years	Total
**Evacuation status**					
Routine	36 (16.4)	75 (46.3)	53 (39.9)	120 (69.4)	284 (41.3)
Priority	125 (57.1)	75 (46.3)	69 (51.9)	46 (26.6)	315 (45.9)
Urgent	58 (26.5)	12 (7.4)	11 (8.3)	7 (4.1)	88 (12.8)
**Daignoses**					
Abdominal	31 (14.2)	16 (9.9)	16 (12)	21 (12.1)	84 (12.2)
Burn	1 (0.5)	6 (3.7)	4 (3)	1 (0.6)	12 (1.8)
Cardiac	50 (22.8)	18 (11.1)	8 (6)	7 (4)	83 (12.1)
Endocrine	3 (1.4)	1 (0.6)	18 (13.5)	22 (12.7)	44 (6.4)
Hematologic/Oncological	7 (3.2)	11(6.8)	31 (23.3)	15 (8.7)	64 (9.3)
Infectious disease	9 (4.1)	10 (6.2)	3 (2.3)	5 (2.9)	27 (3.9)
Neurological	16 (7.3)	17 (10.5)	9 (6.8)	8 (4.6)	50 (7.3)
Orthopedic	0 (0)	0 (0)	6 (4.5)	21 (12.1)	27 (3.9)
Prematurity	39 (17.8)	25 (15.1)	0 (0)	0 (0)	64 (9.3)
Psychiatric	0 (0)	0 (0)	0 (0)	49 (28.3)	49 (7.1)
Respiratory	26 (11.9)	26 (16.1)	19 (14.3)	9 (5.2)	80 (11.6)
Other **Ventilator status**	37 (16.9)	32 (19.8)	19 (14.3)	15 (8.7)	103 (15)
Ventilated	75 (34.3)	29 (17.9)	12 (9)	2 (1.5)	118 (17.2)

All values expressed as *N* (%); % represents the percentage within the age-group.

Table II describes the primary diagnosis category across all patients as well as by age-group. Across all patients, primary transport diagnoses most commonly fell into three categories: abdominal (12.2%, *n* = 84), cardiac (12.1%, *n* = 83), and respiratory (11.6%, *n* = 80). fifteen percent (*n* = 103) of the patients were categorized as “other,” which was reserved for conditions not otherwise classified such as renal, metabolic, and genetic conditions or obstetrical and surgical conditions.

Across all transported patients, 118 (17.2%) were mechanically ventilated during transport. The highest percentage of this cohort were neonatal patients (<31 days), which represented 75 of the 118 intubated patients (63.6%). Those 75 patients comprise 34.3% of all reported neonatal transports. The percentage of patients mechanically ventilated continued to decrease with age. Only two patients aged 8 years and older required mechanical ventilation during transport.

## DISCUSSION

This study reports the first demographic analysis of TRAC2ES data for pediatric transports within INDOPACOM, which is also the first analysis of transoceanic pediatric transports. This has implications for resource allocation, including personnel and equipment, especially given the large proportion of neonatal and infant transports.

It is important to identify that patient needs across different age-groups vary. For example, the largest cohort of patients transported were less than 31 days old with the most common diagnoses that could be readily categorized beyond prematurity being cardiac, respiratory, or abdominal. In contrast, the second largest cohort was patients 8 years and older, and the largest diagnosis category observed within this population was psychiatric, which suggests most of the children in this age category were likely otherwise healthy. It is also notable that the majority of children under age of 8 years were transported in either urgent or priority status, while the majority of those over 8 years had a routine transport status.

Of significant importance, 17.2% of all movements requi-red mechanical ventilation, most of which were neonatal. This corresponds with the high number of patients with a primary respiratory diagnosis. This represents a key finding with regard to staffing of the transport teams and training. Most medical flight personnel come from emergency department and adult ICU backgrounds, which may create a lack of significant exposure to pediatric airway management or ventilator experience (particularly with this age-group) and Pediatric Advanced Life Support (PALS) and Neonatal Resuscitation Program (NRP) are not required for AE or CCATT members.^10^ No pediatric critical care trained personnel, nursing or physician, are assigned, or routinely available for pediatric transport in the INDOPACOM region.^11^ This has implications on transport team training and preparation. For example, when compared to adult airways, pediatric airways are typically more anterior, have a smaller diameter, have a funnel-shaped larynx, and are shorter in length. These anatomic differences can make intubation more challenging for someone inexperienced in managing pediatric airways. Additionally, managing ventilators can be vastly different in adults, children, and neonates who have different physiological needs and response to stressors such as hyperoxia and sedation.

It is observed that the majority of transports originated from Japan, which is the primary location of international U.S. forces in INDOPACOM (outside of Hawaii).^12^ Furthermore, the majority of transports terminated in Hawaii with a smaller percentage ending in the continental United States. The transoceanic transports performed to, from, and within INDOPACOM highlight the unique nature of military pediatric transport. Within the United States, less than one third of neonatal transport teams move patients greater than 500 miles.^7^ A study evaluating transports in remote locations within Canada found that even long-range pediatric transports averaged only 383 km (238 miles.)^13^ In contrast, the flight from Okinawa, Japan to Honolulu, Hawaii is approximately 4,600 miles. Additionally, military pediatric transport within INDOPACOM is unique in that the overwhelming majority of transports (greater than 99%) are performed using fixed-wing aircraft. Within the civilian literature, the capability to use rotary and/or fixed wing aircraft by transport teams is far from universal, with ranges from 20.5% to 74.5%.^7,8^

Generally, within INDOPACOM, patients weighing less than 5 kg or 30 days old were transported by a neonatal team which consisted of a NICU nurse and technician, with a neonatologist as needed. The neonatal team also routinely transported NICU patients older than 30 days but less than 6 months and weighing less than 5 kg who required more definitive care. While a neonatologist could potentially be used in the transport of non-NICU infants older than 30 days but less than 6 months, generally those patients not under the care of the NICU would be transported without a pediatric specialist. Patients not transported by the neonatal team were transported either by an AE team or, if critically ill or requiring advanced airway support, a CCATT. Rarely, missions may have a pediatric specialist on board, if available. However, pediatric specialists, including pediatric intensivists, are not part of the composition of AE teams or CCATT nor do the MTFs have adequate specialized pediatric staff to augment the teams, and thus the majority of patients outside of those coming from the NICU are transported without a pediatric specialist.^11^ The medical controller for transports, including pediatric patients, is a flight surgeon who typically coordinates pediatric transports in conjunction with the sending and/or receiving pediatricians. The increased number of urgent and priority transports in all children under 8 years (not just neonates) suggests higher acuity and more intensive needs in younger pediatric patients. While children who reach puberty can be managed using advanced cardiac life support in emergent situations and have anatomy more closely resembling adults, prepubertal children are managed using PALS (or NRP in the neonate) and have anatomic and physiological differences that require specialized training. More research into increasing the pediatric staffing at specialized MTFs in the region to allow for augmentation of adult teams could be considered.

Within the civilian literature, a previous single-center study demonstrated improved outcomes in neonatal and pediatric transfers conducted by specialized pediatric teams when compared to standard transport teams.^14^ A similar benefit has been seen with specially trained teams in the combat setting; in response to improving outcomes in combat, the U.S. Army has developed critical care trained transport teams within combat theaters.^15^ CCATT physicians and nurses may have pediatric experience if they are trained in emergency medicine but may lack familiarity if their previous experience is limited to adult critical care. Such considerations should be extended to areas requiring special transport as part of the noncombat mission, including pediatric transport. Further research evaluating the ability of CCATT team members, previous training and experience may provide data to support the intelligent tasking of CCATTs with pediatric-trained personnel to transport pediatric patients.

Approximately 98% of civilian neonatal transport teams in the United States require NRP certification, and most pediatric teams require PALS (92%) and NRP (86%.)^16^ Beyond that, training is highly variable and there is no reported standardized training on in-flight physiology.^16^ Unlike civilian transport teams, the training of both AE teams and CCATT is highly regimented.^10,16^ Both teams receive extensive training regarding the transport of patients and have a robust curriculum teaching in-flight physiology.^5,6^ In this regard, both AE teams and CCATT are well equipped to understand and respond to in-flight changes in the clinical status of their patients. However, almost all their training is specific to the operational needs of CCATT, which is generally limited to military-aged personnel as well as contractors in theater.^17^ Very little of the military medical flight training addresses the unique physiology of neonatal and pediatric patients. Military neonatal transport teams are required to have NRP as well as the STABLE course, although these teams are not CCATT trained and thus are not guaranteed to have flight physiology training.^10^ Neither PALS nor NRP are required for CCATT that transport pediatric patients.^10^ This inconsistency highlights a potential area for future training of both AE and CCATT. Further studies may help to determine training and composition gaps for the current CCATT and provide insight regarding ways to better prepare these teams to transport pediatric and neonatal patients. While the composition of the teams itself need not necessarily change, the teams should be adequately trained to care for any patient they may transport.

The study analysis has several limitations. Patients transported by ground to local civilian hospitals would not be captured within TRAC2ES even if transported by a military team. This contributes to an underestimation of the complex and critically ill pediatric transports military medical professionals are tasked with managing. Moreover, there are no data on patients who were too unstable for the long-distance transport and were sent to local civilian healthcare systems. Data entered into TRAC2ES are often done by nonmedical personnel not directly involved in patient care, which may be a potential source of error in the data. The analysis is also limited by the inability to enter more than one patient diagnosis, which could underestimate certain diagnosis categories. For instance, a patient labeled as cardiac based on primary diagnosis may also be a preterm infant, or vice versa. Additionally, there is no consistency to which patient data are entered into the free text history section. The free text boxes often are the only location where information such as transport team (CCATT vs AE), ventilator status may be entered. Given the limitation of free text data, this analysis also likely underestimates the number of patients requiring critical care physician support while in flight. Additionally, there is no definitive record of neonatologists or pediatricians on the transports, although per expert report, there have only been two pediatric transports in INDOPACOM within the past 5 years with a pediatric intensivist onboard.^11^ Due to the variable nature of information entered into the free text of TRAC2ES, invasive ventilation was used as a marker of critical care status, and the data were searched only for patients requiring invasive ventilation. This does not capture patients who may have been transported on noninvasive ventilation such as continuous positive airway pressure or high-flow oxygen, or who may have required cardiac monitoring for medications administered during flight but that were otherwise stable on room air. Finally, due to the limited nature of data entered into TRAC2ES, it is not possible to present outcomes of data regarding adverse events that may have occurred during transport.

This analysis provides the first evaluation of pediatric transports within INDOPACOM recorded in TRAC2ES over the designated 10-year period. The wide variety of patient ages and diagnoses highlights a unique challenge of pediatric transports. While all members of AE teams and CCATT are highly trained in caring for patients in flight, current literature supports ensuring that any crew member caring for pediatric patients has pediatric specific training. Given the volume of military transports of neonatal and pediatric patients in INDOPACOM, investigation into the appropriate composition of INDOPACOM transport teams is warranted. Furthermore, the percentage of urgent and priority transports highlight the need for providers capable of supporting critically ill pediatric patients across the globe.

## CONCLUSIONS

Within TRAC2ES, nearly one in six encounters were patients <18 years old, with neonates or infants representing nearly one of three pediatric encounters. Slightly more than one in six pediatric patients required intubation for transport. The data suggest the need for appropriately trained transport teams and equipment be provided to support these missions.
